# Molecular investigation on hazelnut-associated *Fusarium* isolates belonging to the *Fusarium citricola* species complex

**DOI:** 10.3389/fmicb.2025.1741069

**Published:** 2026-01-09

**Authors:** Federico Brugneti, Silvia Turco, Nepomuscene Ukwibishaka, Barbara Abramczyk, Antonella Cardacino, Angelo Mazzaglia, Rosario Nicoletti, Luigi De Masi, Beata Zimowska

**Affiliations:** 1 Department of Agriculture and Forest Science, Tuscia University, Viterbo, Italy; 2 Department of Plant Protection, Subdepartment of Phytopathology and Mycology, University of Life Sciences, Lublin, Poland; 3 Research Centre for Olive, Fruit and Citrus Crops, Council for Agricultural Research and Economics, Caserta, Italy; 4 Institute of Biosciences and BioResources, National Research Council, Portici, Italy

**Keywords:** *Fusarium citricola* species complex, *Fusarium tricinctum* species complex, hazelnut mycobiome, phylogenesis, species delimitation analysis

## Abstract

Recent studies on *Fusaria* associated with hazelnut have pointed out a role of these fungi as both disease agents and endophytic symbionts, raising concern for the possible mycotoxin contamination of kernels and derived products. Molecular evidence has shown that previous classifications of these isolates as *Fusarium lateritium* were incorrect, indicating that most of them instead belong to the *Fusarium citricola* species complex (FCCSC). Based on a set of isolates collected in Italy and Poland, the present work provides a phylogenetic analysis supported by three species delimitation algorithms. The results confirm that all the available hazelnut isolates belong to the FCCSC, and that the discrimination between three currently accepted taxa in this species complex, namely *F. aconidiale, F. celtidicola* and *F. juglandicola*, should be reconsidered. The inclusion in our analysis of 25 species identified in the closely related *Fusarium tricinctum* species complex provides an indication that the statistical methods for species delimitation represent a useful tool for checking the reliability of the species boundaries currently defined in these fungi.

## 1 Introduction

The genus *Fusarium* comprises a large and diverse group of filamentous ascomycetous fungi that are ubiquitous in nature, colonizing soil, plant debris, and a variety of other substrates. Many *Fusarium* species are recognized as major plant pathogens, causing devastating diseases across a wide range of crops worldwide (Todorović et al., 2023). Recent taxonomic studies have revealed that the genus *Fusarium* comprises more than 400 phylogenetically distinct species, grouped into over 30 species complexes and several monotypic lineages (Hof and Schrecker, 2024). These advances, together with ongoing revisions based on molecular data, continue to reshape the taxonomy of the genus through the description of new species and the redefinition of phylogenetic relationships (Crous et al., 2022; O’Donnell et al., 2022; Ulaszewski et al., 2025). Although morphological features provide valuable diagnostic information, their reliability for species-level identification in *Fusarium* is limited by high phenotypic plasticity and overlapping traits among species, often influenced by culture conditions and environmental factors. This morphological convergence, or synapomorphy, where distantly related taxa share similar characters, has historically complicated *Fusarium* taxonomy and the species identification (Stępień, 2014; Manganiello et al., 2019; Crous et al., 2022). The integration of molecular approaches, particularly DNA barcoding and multilocus sequencing, has therefore become essential for accurate species delineation, providing greater resolution within cryptic or recently diversified lineages (Summerell, 2019; Sandoval-Denis et al., 2018; Lombard et al., 2021; Stoeva et al., 2023; Kamil et al., 2025).

The economic impact of hazelnut (*Corylus avellana*) is increasing worldwide, with an estimated global production of 1.15 million tons in 2023; Turkey is the leader country, producing as much as 56% of shelled hazelnuts, followed by Italy with a market share of about 9% (FAOSTAT; www.fao.org/statistics/en, accessed on 30 November 2025). A major concern for product storage and marketing is represented by fungi causing kernel contamination with a wide array of mycotoxins, for which there is increasing attention by the control authorities (Salvatore et al., 2023). In this respect, the role by *Fusarium* spp. deserves more-in-depth assessments in light of their reputation as producers of mycotoxins, such as trichothecenes and enniatins, and the increasing number of reports in association with this crop (Munkvold et al., 2021; Gautier et al., 2020; Zimowska et al., 2024). Indeed, *Fusaria* isolated from hazelnut represent a meaningful example of how traditional morphology-based taxonomic schemes are insufficient for accurate species identification and have often led to misclassification. Early investigations on the nut gray necrosis (NGN) affecting hazelnuts in the Viterbo area (Central Italy) carried out in the first decade of the 2000s identified *Fusarium lateritium* as the causal agent (Vitale et al., 2011). However, genomic evidence from a more recent study questioned this identification, highlighting a closer affinity of these isolates with the *Fusarium tricinctum* species complex (FTSC) (Turco et al., 2021). Following the characterization of a new lineage related to the FTSC, designated as *Fusarium citricola* species complex (FCCSC) (Sandoval-Denis et al., 2018), two endophytic isolates from hazelnut collected in Poland were identified as members of this complex (Zimowska et al., 2024). Although this inference was later confirmed after the whole genome sequencing of one of these isolates (Becchimanzi et al., 2025), the limited number of reference strains available at that time did not allow to conclusively assign them to either *Fusarium celtidicola* or *Fusarium juglandicola*. Similar uncertainty regarding the separation between these species also emerged in a recent phylogenetic study based on strains available from reputed mycological collections, which had been classified as *F. lateritium* and found to rather belong to either the FTSC or the FCCSC (Costa et al., 2024). In this context, the availability of a broader set of isolates from both Italy and Poland enabled us to perform a more comprehensive phylogenetic reconstruction supported by species delimitation analyses. These species delimitation methods provide a valuable tool for resolving complex taxonomic relationships and detecting cryptic diversity, as demonstrated in other Ascomycota lineages (Liu et al., 2016; Bustamante et al., 2019; Maharachchikumbura et al., 2021; Becchimanzi et al., 2021; Sklenář et al., 2022; Dissanayake et al., 2024; Zapata et al., 2024). In this framework, the present study aims to clarify the taxonomic position of *Fusarium* isolates associated with hazelnut and to evaluate the validity of the current species boundaries within the *F. citricola* species complex (FCCSC).

## 2 Materials and methods

### 2.1 *Fusarium* isolates

In May and June 2023, a field survey was carried out in three hazelnut orchards located in the Viterbo area (Central Italy), to investigate the presence of early NGN symptoms. Twenty five symptomatic hazelnut samples, collected from cultivar ‘Nocchione’ and ‘Tonda Romana’, were sealed in sterile plastic bags and brought to the Plant Pathology Laboratory of the Tuscia University in Viterbo. Briefly, hazelnut inner kernel sections were surface-sterilized with 3% sodium hypochlorite for 3 min, rinsed twice with sterile distilled water, and dried under laminar flow. One-centimeter sections from both healthy and symptomatic tissues were aseptically placed onto potato dextrose agar (PDA, Dinkelberg analytics, Gablingen, Germany) plates and incubated at 25 ± 1 °C for 5–7 days. The pure fungal cultures were obtained through serial transfer of emerging colonies onto fresh PDA plates and further used for morphological and molecular analyses. For morphological characterization, single hyphal tips were transferred to plates containing the standard PDA and oatmeal agar (OA, Condalab, Madrid, Spain), which were incubated at 25 °C in the dark. After 10 days, the morphology of the colony was observed. Morphology of the conidia was examined by growing the isolates on synthetic nutrient agar (SNA), prepared according to Nirenberg (1976). Plates were incubated at 25 °C in the dark at 100% relative humidity. Observations on the conidial morphology and size were carried out at 100x magnification through a microscope (Leitz).

In addition to those available from our previous study (Zimowska et al., 2024), four more endophytic isolates conforming to the FCCSC were collected from secondary branches of both hazelnut and small-leaved linden (*Tilia cordata*) in the Kraków area (Southwest Poland), following the procedure described in the mentioned reference. The details concerning the isolation sources and the GenBank accession numbers of the DNA barcode sequences used in this study are indicated in Table 1.

**Table 1:** *Fusarium* isolates obtained in this study and their DNA barcode sequence GenBank accession numbers.

Strain number	Origin	*tef1*	*rpb2*
FUS 3	*Corylus avellana* NGN, Italy	PX244362	PX244345
FUS 4	*C. avellana* NGN, Italy	PX244363	PX244346
FUS 7	*C. avellana* NGN, Italy	PX244364	PX244347
FUS 8	*C. avellana* NGN, Italy	PX244365	PX244348
FUS 11	*C. avellana* NGN, Italy	PX244366	PX244349
FUS 12	*C. avellana* NGN, Italy	PX244367	PX244350
FUS 13	*C. avellana* NGN, Italy	PX244368	PX244351
FUS 14	*C. avellana* NGN, Italy	PX244369	PX244352
FUS 15	*C. avellana* NGN, Italy	PX244370	PX244353
FUS 16	*C. avellana* NGN, Italy	PX244371	PX244354
FUS 18	*C. avellana* NGN, Italy	PX244372	PX244355
FUS 19	*C. avellana* NGN, Italy	PX244373	PX244356
FUS 21	*C. avellana* NGN, Italy	PX244374	PX244357
FUS 22	*C. avellana* NGN, Italy	PX244375	PX244358
FUS 23	*C. avellana* NGN, Italy	PX244376	PX244359
FUS 24	*C. avellana* NGN, Italy	PX244377	PX244360
FUS 25	*C. avellana* NGN, Italy	PX244378	PX244361
Hzk 18	*C. avellana* endophyte, Poland	PX521117	PX521113
Hzk 28	*C. avellana* endophyte, Poland	PX521118	PX521114
Ti 3	*Tilia cordata* endophyte, Poland	PX521115	PX521111
Ti 17	*T. cordata* endophyte, Poland	PX521116	PX521112

### 2.2 DNA extraction and PCR amplification for molecular characterization

Genomic DNA (gDNA) was extracted from fresh, filtered mycelium obtained from pure colonies grown in 50 mL tubes containing potato dextrose broth (PDB) using the Plant Genomic DNA Extraction Mini Kit (Fisher Molecular Biology, Rome, Italy). Molecular characterization was carried out following published protocols (O'Donnell et al., 1998; Liu et al., 1999; Hofstetter et al., 2007), through amplification of the translation elongation factor 1-alpha (*tef-1*) and the second largest subunit of RNA polymerase II (*rpb2*) gene regions, using the primers listed in Table 2. For each PCR reaction, 5 ng of gDNA was used as a template in a final volume of 25 μL, containing 2 × PCRBIO HS Taq DNA Polymerase (PCR Biosystems, UK) and 0.5 μM of both forward and reverse primers. The thermal cycling program for *tef-1*, using primers EF-1 and EF-2, consisted of an initial denaturation at 94 °C for 3 min, followed by 35 cycles of denaturation at 94 °C for 30 s, annealing at 55 °C for 30 s, and extension at 72 °C for 30 s, with a final extension at 72 °C for 5 min. For *rpb2* amplification, using primers fRPB2-5f and fRPB2-7cR, the program consisted of an initial denaturation at 94 °C for 5 min, followed by 35 cycles of denaturation at 94 °C for 60 s, annealing at 57 °C for 75 s, and extension at 72 °C for 60 s, with a final extension at 72 °C for 10 min. Two μL of the amplified products were analyzed by 1.2% agarose gel electrophoresis, while the remaining products were sent to Macrogen Europe (Milan, Italy) for Sanger sequencing.

**Table 2:** Primers used for the molecular characterization of the *Fusarium* isolates.

Primer	Sequences 5’–3’	Reference
EF-1	5’-ATGGGTAAGGA(A/G)GACAAGAC-3’	O'Donnell et al. (1998)
EF-2	5’-GGA(G/A)GTACCAGT (G/A)ATCATGTT-3’	O'Donnell et al. (1998)
fRPB2-5f	5’-GAYGAYMGWGATCAYTTYGG-3’	Hofstetter et al. (2007)
fRPB2-7cR	5’-GTA(A/G)TTCAT(C/T)AC(A/G)CCNGG-3’	Liu et al. (1999)

### 2.3 Phylogenetic and species delimitation analyses

All the collected *Fusarium* isolates were submitted to a detailed phylogenetic analysis, along with 60 strains belonging to the FCCSC and the FTSC whose sequences are available in the GenBank database (Supplementary Table 1); *F. lateritium* NRRL 13622 was included as an outgroup. The original sequences were edited using UGENE v48.1 (Okonechnikov et al., 2012), concatenated, and aligned with MUSCLE v3.8.31 (Edgar, 2004). The resulting alignment file was used as input to construct a maximum likelihood (ML) phylogenetic tree with RAxML-HPC v8.2.12 (Stamatakis, 2014), employing the GTRGAMMAI substitution model and 1,000 bootstrap replicates. The tree was visualized with FigTree v1.4.4^1^ and further edited with Inkscape v0.92.^2^

Species delimitation analysis was performed employing the distance-based Automatic Barcode Gap Discovery (ABGD), the Generalized Mixed Yule-Coalescent (GMYC) model, and the Poisson Tree Processes method with multi-rate (mPTP) implementations, using RAxML output tree as input. In particular, the ABGD algorithm splits sequences based on break points in pairwise genetic distances (“barcode gaps,” Puillandre et al., 2012). The mPTP algorithm (Kapli et al., 2017) inspects a substitution-based, non-ultrametric phylogenetic tree to detect changes in branching rates. These rates, modeled as a Poisson process, occur randomly at a relatively constant rate within species and more slowly between species, allowing the algorithm to delineate species boundaries. The GMYC algorithm uses a time-calibrated (ultrametric) tree to find the point where the branching pattern shifts from splits between species to merge among individuals of the same species; thus, it is really sensitive to recent divergence when they tend to over-split. For the ABGD analysis, the parameters were set to test variability (P) between 0.001 (Pmin) and 0.1 (Pmax), standard for fungal ITS or protein-coding markers (Puillandre et al., 2012), with a minimum gap width of 0.1, employing the Kimura 2-parameter model and 50 screening steps. For the mPTP approach, 50 million generations were employed with MCM or ML algorithm, with sampling every 1,000 generations, using the minimum branch length calculated from each tree. For the GMYC analysis, an ultrametric tree was constructed on BEAST2 v2.7.7 (Bouckaert et al., 2014), setting the gamma site model with GTR + G + I as substitution, the Yule prior with a relaxed molecular clock, and Markov Chain Monte Carlo (MCMC) run for 50 million generations, sampled every 1,000 generations. Convergence and effective sample size (ESS) higher than 200 was checked using Tracer v.1.7.2 (Rambaut et al., 2018) and the maximum credibility clade tree was obtained with Treeannotator v2.7.7 (Bouckaert et al., 2014) applying the mean heights parameter and discarding the first 10% of the trees as burn-in period. The resulting tree was then imported into the R environment and the GMYC analysis was performed using the *splits* package using the single threshold approach (Fujisawa and Barraclough, 2013).

## 3 Results

### 3.1 Morphological features

**Figure 1:**
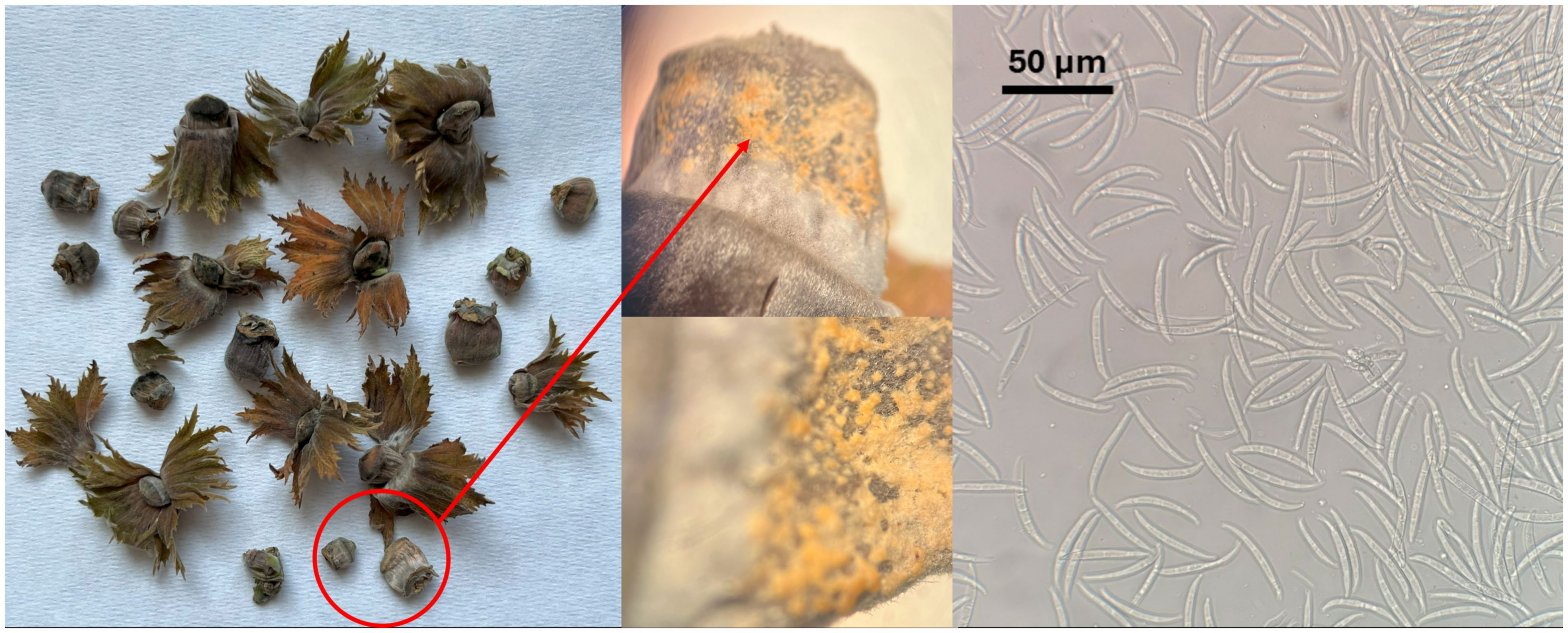
Symptomatic hazelnuts showing *Fusarium* sporodochia and macroconidia.

The hazelnut samples collected in orchards located in the Viterbo area from NGN symptomatic plants showed the presence of orange to light brown sporodochia on the fruit surface, indicating active fungal growth; these fruiting structures contained numerous hyaline, multiseptate, crescent-shaped macroconidia (Figure 1). The isolations done from the inner kernel tissues consistently yielded *Fusarium* from all the samples examined; a total of 17 isolates were recovered and stored in pure culture for the subsequent assessments. Four more *Fusarium* isolates exhibiting similar morphological features were recovered in the Kraków area, respectively two from hazelnut and two from linden tree, within the cooperative work in progress concerning endophytic associates of forest trees (Nicoletti and Zimowska, 2023). Notably, no symptoms referable to NGN were observed in the course of inspections carried out in Southern Poland in summer 2025.

**Figure 2:**
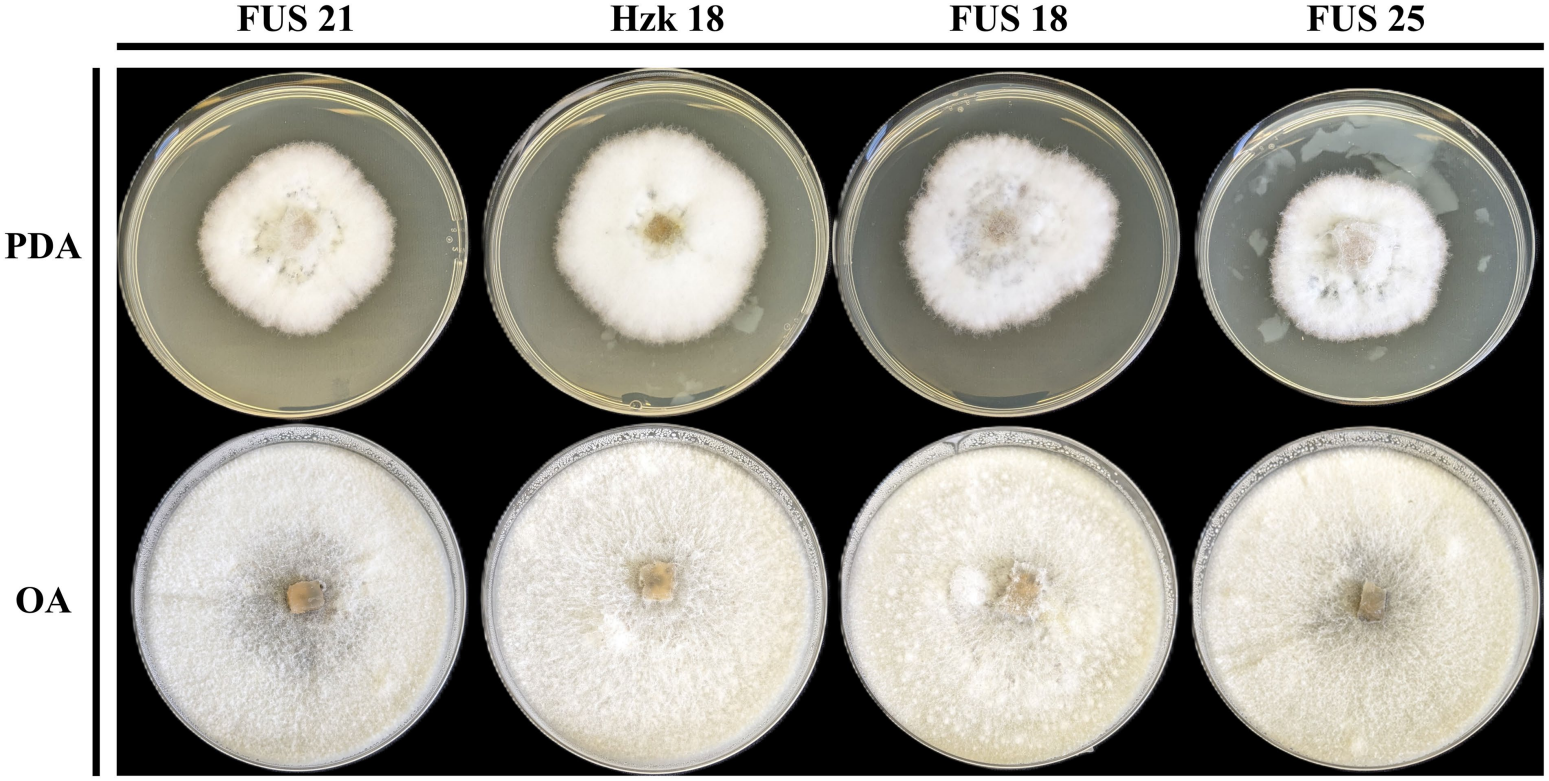
Colony morphology of representative isolates on PDA and OA after 10-day incubation at 25 °C.

**Figure 3:**
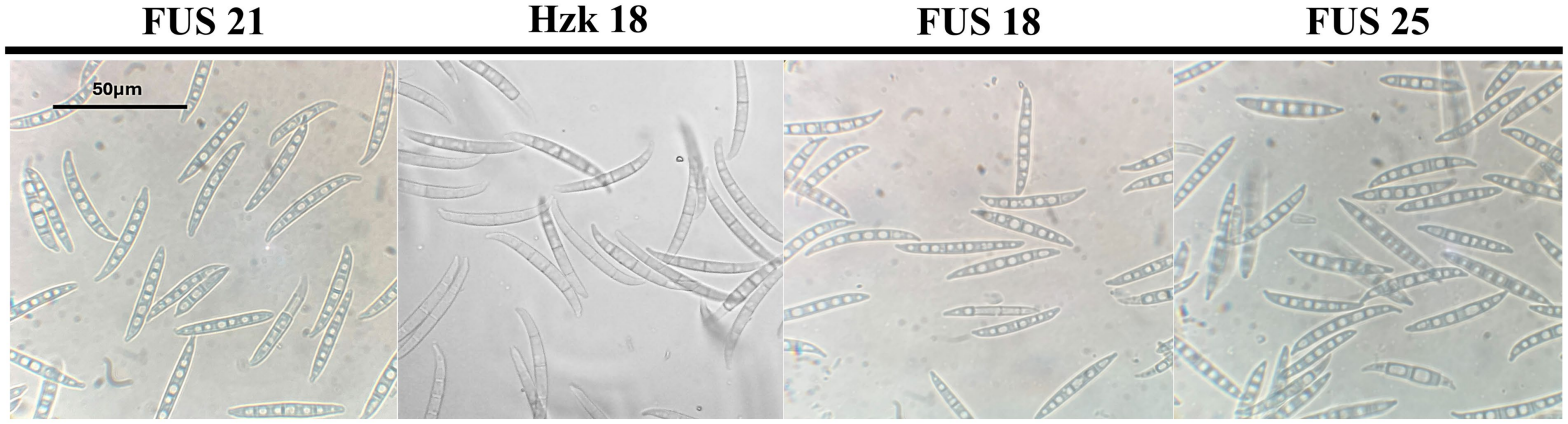
Morphology of macroconidia of isolate FUS 21, Hzk 18, FUS 18, and FUS 25 after 2-week incubation on SNA at 25 °C.

**Figure 4:**
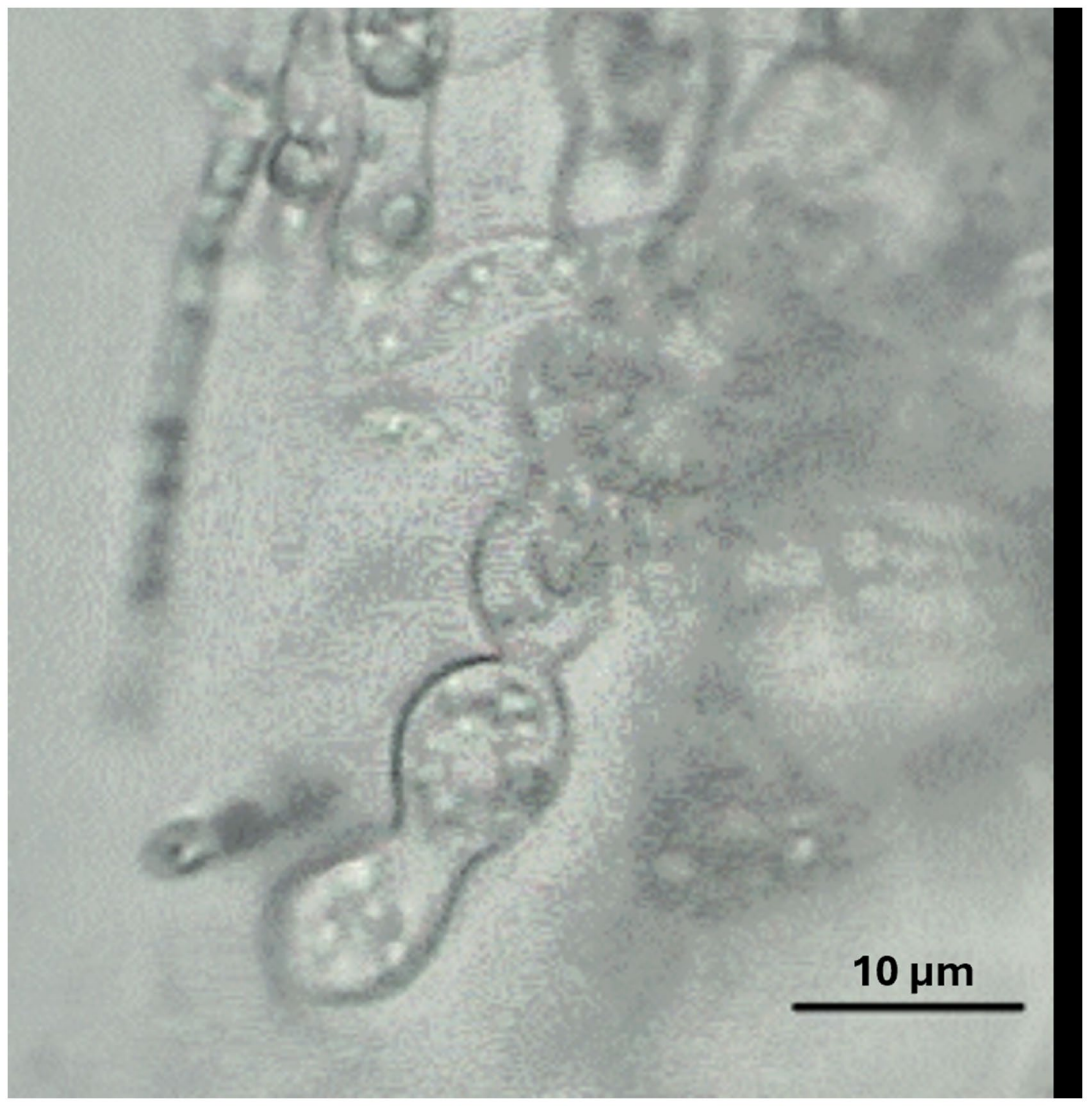
Chlamydospores of isolate Hzk 18 produced on SNA after 2-week incubation at 25 °C. Scale bar = 10 μm.

When grown in axenic culture on PDA, the morphological characteristics of both pathogenic and endophytic isolates were comparable and consistent with the description previously reported for Polish isolates (Zimowska et al., 2024). On OA, radial growth was more pronounced; however, sporulation was generally reduced, and only minor differences in colony color and morphology were observed (Figure 2). On SNA, abundant macroconidia were produced from monophialidic conidiogenous cells. These conidia were hyaline, multicellular, typically with three to five septa, slightly curved and tapering toward both ends. The apical cells were more distinctly curved, whereas the basal cells exhibited a characteristic foot-shaped morphology (Figure 3). Throughout the 14-day incubation period, no pigmentation of the medium was detected, and microconidia were not produced. Chlamydospores were absent in the Italian isolates, whereas they were consistently observed in all the Polish isolates examined (Figure 4). The relevant morphological features as assessed in comparison with the descriptions of the reference FCCSC species, and reported for homogeneous groups of isolates, are resumed in Table 3.

**Table 3:** Main morphological characters of the groups of isolates delimited in the phylogenetic analysis in comparison with the related FCCSC species.

Species/strains	Colony description	Macroconidia	Microconidia	Clamydospores
Hzn1, Hzn5	Colony Ø after 7 days on PDA: 37 mm; on OA: 75 mm; aerial mycelium on PDA abundant and dense, floccose to wooly with crenate margin, white-cream; reverse salmon-orange. Dark gray sclerotia-like structures visible in 30-day old cultures	Mostly 3–4 septate, hyaline, unequally curved; apex pointed, base foot-shaped, poorly developed. Abundantly formed at monophialidic conidiogenous cells	Absent	Chlamydospores abundant, formed quickly mainly in chains, but also single or paired, smooth-walled, intercalary, globose-subglobose to pyriform
FUS 3, FUS 11, FUS 13, FUS 14, FUS 15, FUS 21, FUS 23, FUS 24	Colony Ø after 7 days on PDA: 34 mm; on OA: 81 mm; aerial mycelium on PDA dense, floccose to wooly with irregular margin, white-cream; reverse, light salmon with dark areas in the center. On OA abundant white floccose aerial mycelium produced with dark sclerotia, cream colored on the back	Abundantly formed at monophialidic conidiogenous cells on SNA, hyaline, with an average of 4–5 septa, slightly curved, tapered at the ends; apical cells more curved, basal cells foot-like shaped	Absent	Absent
Hzk 18, Hzk 28, Ti 3, Ti 17	Colony Ø after 7 days on PDA: 35 mm; on OA: 80 mm; aerial mycelium on PDA dense, floccose to wooly with irregular margin, white-cream; reverse salmon-orange. Dark gray sclerotia-like structures visible in 30-day old cultures	Mostly 4-5 septate, hyaline, slightly curved; apex pointed, base foot-shaped. Abundantly formed at monophialidic conidiogenous cells	Absent	Chlamydospores formed in older cultures mainly in chains, but also single or paired, smooth-walled, intercalary, globose or subglobose
FUS 4, FUS 7, FUS 8, FUS 12, FUS 16, FUS 18, FUS 19, FUS 22	Colony Ø after 7 days on PDA: 34 mm; on OA: 81 mm; aerial mycelium on PDA dense, floccose to wooly with irregular margin, white-cream; reverse, light salmon with dark areas at the center. On OA abundant white floccose aerial mycelium produced with dark sclerotia, cream colored on the back	Abundantly formed at monophialidic conidiogenous cells on SNA, hyaline, with an average of 3-4 septa, slightly curved, tapered at the ends; apical cells more curved, basal cells foot-like shaped	Absent	Absent
FUS 25	Colony Ø after 7 days on PDA: 32 mm; on OA: 79 mm; aerial mycelium on PDA dense, floccose to wooly with irregular margin, white-cream; reverse, light salmon with dark areas at the center. On OA abundant white floccose aerial mycelium produced with dark sclerotia, cream colored on the back	Abundantly formed at monophialidic conidiogenous cells on SNA, hyaline, with an average of 4 septa, sporadically with 5 septa, slightly curved, tapered at the ends; apical cells more curved, basal cells foot-like shaped	Absent	Absent
*Fusarium celtidicola* (Shang et al., 2018)	Colony Ø after 7 days on PDA: 2.5–4 mm; aerial mycelium on PDA white to yellowish or vinaceous, edge crenate, flat or effuse. Colonies white above, reddish at the center, reverse reddish-white	3–5-septate, hyaline, naviculate to falcate, beak at the base	Absent	Chlamydospores ellipsoidal to pyriform, single or chain inter the hyphae
*Fusarium juglandicola* (Crous et al., 2022)	Colonies on PDA white to pale luteous on surface and reverse	(1–)3–4(–5)-septate, falcate, moderately dorsiventrally curved with almost parallel sides, tapering toward the ends, with a blunt to slightly hooked, somewhat curved apical cell and papillate to well-developed, foot-shaped basal cell	Absent	Absent
*Fusarium aconidiale* (Crous et al., 2022)	Colonies on PDA white to rosy buff, flat, wooly to cottony with radial patches of white aerial mycelium, reverse white to pale rosy buff	3(–5)-septate, falcate, straight to moderately curved, tapering toward the base, apical cell equally sized than the adjacent cell, curved to hooked; basal cell well developed, foot-shaped, rarely papillate	Absent	Absent

### 3.2 Phylogenetic and species delimitation analyses

**Figure 5:**
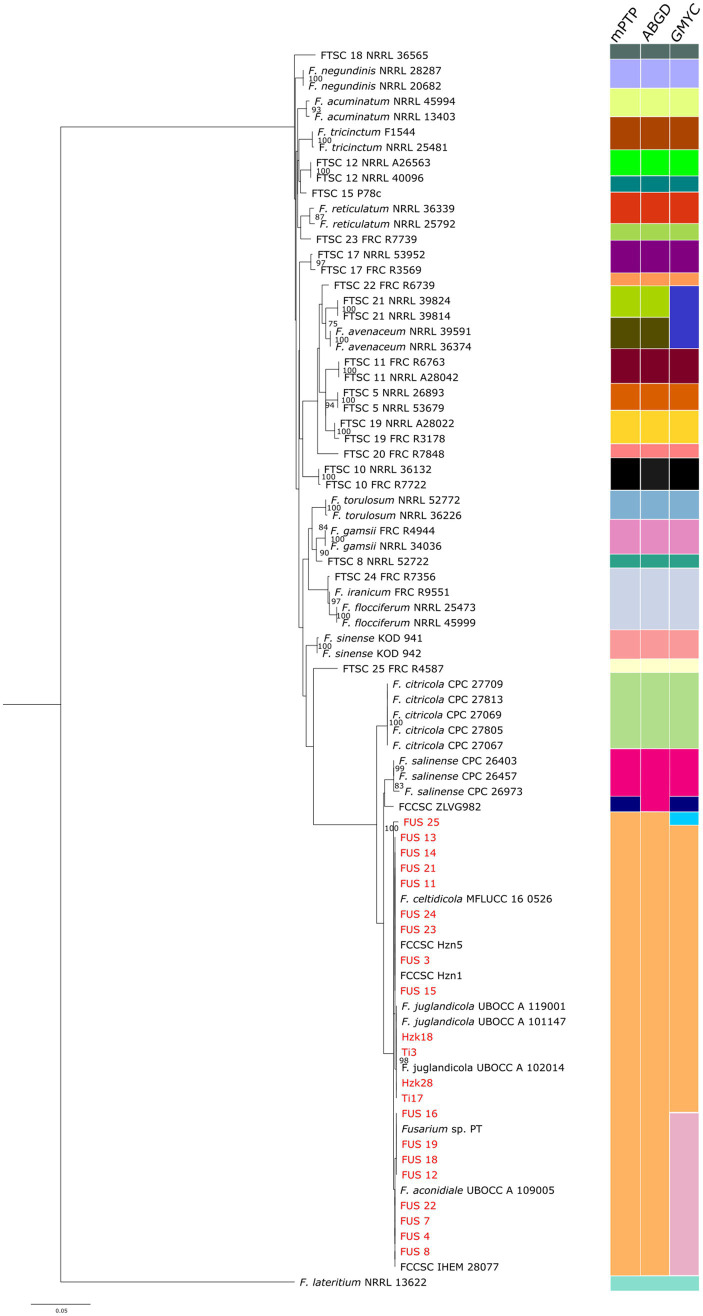
Species delimitation of the combined *tef-1* and *rpb2* sequences of 81 *Fusarium* isolates according to ABGD, mPTP, and GMYC algorithms. Bootstrap values indicating the robustness of the clustering are reported as node values ≥70.

The phylogenetic analysis based on the selected DNA markers (Figure 5) confirmed that all strains analyzed belong to the *Fusarium citricola* species complex (FCCSC), although they exhibit a certain degree of intra-clade variation. The 17 strains collected from the Viterbo area are positioned in the lower portion of the dendrogram and form four main clusters. The first cluster consists exclusively of isolate FUS 25; the second includes the type strain of *F. aconidiale* together with isolate PT, previously assigned to the FTSC (Turco et al., 2021); the third group includes *F. celtidicola* and the endophytic isolates from the Lublin area; and the fourth occupies an intermediate position, encompassing *F. juglandicola* and the isolates from the Kraków area. As a whole, the FCCSC and its related isolates are clearly separated from the rest of the strains, reinforcing their distinction from the FTSC.

This separation is consistently supported by all the three independent species-delimitation algorithms (Figure 5). In fact, both mPTP and ABGD assigned all the 21 isolates to a single species-level unit, whereas GMYC identified three distinct entities. The first GMYC group comprised a single NGN isolate (FUS 25), which forms an independent lineage congruent with its unique placement in the phylogenetic tree. The second group included eight Italian NGN isolates, all the Polish isolates, and the strains identified as *F. celtidicola* and *F. juglandicola*. The third cluster encompassed the remaining NGN isolates, including isolate PT, isolate IHEM 28077, and the type strain of *F. aconidiale*. All the three delimitation methods consistently recognized *F. citricola* and *F. salinense* as distinct species, but the placement of isolate ZLVG982 was discordant. In the ABGD analysis this isolate grouped with *F. salinense*, whereas in both mPTP and GMYC it formed an independent lineage, supporting our earlier inference that it may represent a separate, as-yet undescribed species (Zimowska et al., 2024).

Within the FTSC, the three algorithms are generally concordant, consistently recovering well-supported clades corresponding to *F. acuminatum, F. tricinctum, F. torulosum, F. reticulatum, F*. *avenaceum*, and *F. gamsii.* Minor discrepancies concern the discrimination among *F. iranicum*, *F. flocciferum* and FTSC 24, which were grouped as a single taxon by all three delimitation methods, as well as isolate FTSC 21, which clustered together with *F. avenaceum* into a single operational unit in the GMYC analysis. Also noteworthy was the case of FTSC 25, represented by a single strain, which was placed in an intermediate position at a seemingly equal phylogenetic distance from both the FCCSC and the other FTSC lineages. This finding, consistent with our previous phylogenetic results (Becchimanzi et al., 2025), deserves further examination if additional isolates belonging to this provisional taxon become available.

### 3.3 In-depth investigation of K2P genetic distance

Pairwise Kimura 2-parameter (K2P) distance matrices derived from the ABGD analysis confirmed a clear discontinuity between the FCCSC and FTSC species complexes (Supplementary Figure 1). Inter-complex distances ranged from 0.0614 to 0.0955, with a median of 0.0767, delineating a clear barcode gap that separates the two complexes (Supplementary Table 2). By contrast, intra-complex distances were much smaller: within the FTSC, distances were higher (Q3 = 0.0406, i.e., the 75th percentile of the dataset, meaning that 75% of all pairwise distances are smaller than this value and 25% are larger; max = 0.0560), reflecting greater internal structuring associated to several distinct, recognized species-level entities. Within the FCCSC, values remained very low, with a median of 0.0036, Q3 of 0.0229 and max distance of 0.0279 (Supplementary Figure 2; Supplementary Table 2). These results confirm a strong genetic separation between the two complexes, with no overlap between their intra- and inter-group distance distributions. This absence of overlap represents a clear “barcode gap”, quantitatively reinforcing the distinct evolutionary identity of the FCCSC and its separation from the FTSC.

When going deeper into the FCCSC, the K2P distance matrix revealed a cohesive but internally structured lineage composed of four genetic groups: *F. citricola*, *F. salinense*, *Fusarium* sp. ZLVG982, and a larger assemblage including *F. aconidiale*, *F. celtidicola*, *F. juglandicola*, and the Italian and Polish isolates (referred to as “the FCCSC comprehensive taxon, or FCCSC-CT”), in line with our previous phylogenetic consideration. Pairwise distances within these groups were uniformly low, with no variation observed in *F. citricola* (all distances = 0, indicating that these five isolates are genetically identical for the loci analyzed), and slight variability observed in *F. salinense* (median = 0.0048; max = 0.0048) and in FCCSC-CT isolates (median = 0.0024; Q3 = 0.0036, max = 0.0060). Between-group comparison within the FCCSC also yielded very low divergence, with median values ranging from 0.016 to 0.023 K2P (Supplementary Table 2). The smallest inter-group distance was recorded between ZLVG982 and *F. salinense* (median = 0.0144), whereas the largest occurred between *F. citricola* and *F. salinense* (median = 0.0230). The FCCSC-CT consistently showed low differentiation from all the other FCCSC subgroups (median = 0.0168-0.0229). Their internal distances (median = 0.0024) fall well within the expected intraspecific range, while their inter-group divergences (0.017-0.023) suggest incipient speciation rather than established separation. Collectively, these data indicate that the FCCSC-CT constitutes a single, genetically cohesive lineage distinct from, but closely related to, *F. citricola*, *F. salinense* and ZLVG982; together, they form a compact species complex characterized by shallow internal divergence and strong inter-complex separation. Regarding FTSC 25, which is intermediate between FTSC and FCCSC, the distance indicates that this provisional taxon is genetically closer to the former complex. Specifically, the distances from the FTSC were: min distance = 0.0333, max distance = 0.0560, and median = 0.0430; while higher values could be determined toward the FCCSC: min distance = 0.0674, max distance = 0.0739, and median = 0.0700.

## 4 Discussion

The genus *Fusarium* includes a highly diverse assemblage of plant pathogens, endophytes, and saprotrophs occupying a wide range of ecological niches. Its remarkable morphological plasticity and the frequent occurrence of cryptic species have long complicated species delimitation (Summerell, 2019; Crous et al., 2022). Traditional morphology-based identification is often unreliable, as diagnostic traits may vary with culture conditions or overlap among distinct taxa. The introduction of molecular tools has greatly refined *Fusarium* taxonomy, leading to the recognition of several major species complexes through multilocus phylogenetic approaches (O’Donnell et al., 2015; Lombard et al., 2015). However, despite these advances, many recently described species are still based on a limited number of isolates, raising questions about the robustness of their typification and the stability of species boundaries.

Among these, the FCCSC represents one of the most recently delineated and least resolved groups, characterized by low interspecific divergence and overlapping morphological features (Crous et al., 2022; Costa et al., 2024). Within this framework, the present study provides an integrative assessment of FCCSC isolates from Italy and Poland associated with hazelnut, combining morphological observations with phylogenetic inference and three complementary species delimitation methods.

Consistent with previous uncertainties, morphological variation was observed among the analyzed isolates, none of which fully matched the original descriptions of *F. celtidicola*, *F. juglandicola*, or *F. aconidiale* (Shang et al., 2018; Crous et al., 2022). Moreover, the uneven micromorphological traits did not correspond clearly to their phylogenetic distribution. The cluster centered on the type strain of *F. celtidicola* included isolates lacking microconidia and medium pigmentation, with only a few producing chlamydospores. Conversely, the four endophytic isolates associated with *F. juglandicola* all produced chlamydospores, a feature absent from the species’ original description. Finally, the eight isolates related to *F. aconidiale* were most consistent with its morphological profile, although they exhibited macroconidia with a lower average number of septa.

The examination of phylogenetic relationships through genetic distances and species delimitation analyses does not support the current discrimination among these species within the FCCSC. Delimiting species boundaries in recently diversified fungal lineages is inherently challenging, particularly in complexes such as the FCCSC, where low sequence divergence, incomplete lineage sorting, and possible gene flow can obscure the evolutionary patterns, as observed in other *Fusarium* species complex (O’Donnell et al., 2009; Vu et al., 2019). To explore these boundaries, we applied three complementary species delimitation algorithms, ABGD, mPTP, and GMYC, each based on distinct theoretical and evolutionary assumptions. Although they produced partially divergent outcomes, their combined results provide a coherent picture of a genetically cohesive yet internally structured species complex.

The more conservative ABGD and mPTP methods identified a single species-level taxonomic unit including all isolates within the FCCSC, since they failed to detect any significant gap and branching rate shifts within the FCCSC isolates, while clearly discriminating between the two complexes.

Despite being designed to infer species boundaries using a single-locus gene tree, the GMYC model (Fujisawa and Barraclough, 2013) has been extensively used in defining species boundaries in several taxonomic groups using concatenated loci (Liu et al., 2016; Luo et al., 2018; Hilário et al., 2021; Dissanayake et al., 2024; Zapata et al., 2024). Here, GMYC splits the FCCSC-CT into three subgroups, most likely identifying subpopulation structures, rather than real differentiated species. This finer subdivision is consistent with the model’s sensitivity to shallow population-level divergence and has been frequently reported in other *Fusarium* complexes where recent diversification conceals clear species boundaries (Lombard et al., 2015; Crous et al., 2022). The different outcomes among the three algorithms reflect their distinct underlying principles. While the distance- and rate-based methods (ABGD and mPTP) emphasize clear genetic discontinuities, the coalescent-based GMYC is more sensitive to recent divergence and population structure. The slight over-splitting observed with GMYC has similarly been reported in other *Fusarium* complexes such as the *F. incarnatum–equiseti* and *F. tricinctum* s.c. (Marin-Felix et al., 2019; Xia et al., 2019).

Quantitative analyses of K2P distances independently supported the species delimitation results, confirming a clear genetic gap between the FCCSC and the FTSC. Inter-complex distances were more than three times higher than those observed within the FCCSC, underscoring their distinct evolutionary separation.

Within the FCCSC, genetic variation remained minimal: intra-group distances were an order of magnitude lower (median = 0.002–0.005) than the interspecific thresholds typically recognized for *Fusarium* (0.04–0.06; Vu et al., 2019), indicating strong internal cohesion. Four main genetic clusters were identified, *F. citricola*, *F. salinense*, *Fusarium* sp. ZLVG982, and the broad FCCSC-CT assemblage. The latter group displayed very shallow divergence consistent with intraspecific variability rather than distinct speciation, in line with the GMYC results. Isolate ZLVG982 from Slovenia occupied an intermediate position, suggesting an incipient lineage possibly reflecting early divergence or limited geographic isolation. Overall, the concordant outcomes of ABGD and mPTP, supported by low K2P distances, indicate that the FCCSC represents a single monophyletic lineage with shallow but structured intraspecific diversity.

However, as the analyses were based on two loci (*tef1-α* and *rpb2*) and on a defined number of Italian and Polish isolates, additional genomic data, together with an expanded sampling across hosts and regions, will be essential to determine whether the observed variability reflects early speciation or polymorphism within a recently diversified species complex. To the best of our knowledge, the present study was comprehensive of all the strains currently ascribed to the three species in question within the FCCSC. *Fusarium juglandicola* has also been identified in Poland in leaves of mistletoe (*Viscum album* subsp. *austriacum*) (Jankowiak et al., 2023) and on diseased stems of pedunculate oak (*Quercus robur*) seedlings (Jankowiak et al., 2025), as well as in Slovakia on larvae and inside galls of the cecidomyid midges *Asphondylia echii* and *Lasioptera rubi* (Pyszko et al., 2024); unfortunately, the pair of marker sequences required for phylogenetic assessments were not available in GenBank for including these strains in our analyses. Despite these limitations, the close genetic relatedness of isolates collected from both cultivated and wild hosts suggests that members of the FCCSC are widespread and potentially share an endophytic phase in hazelnut. In line with the recent observations by Costa et al. (2024), many strains previously classified as *F. lateritium* likely belong to the FCCSC, reinforcing the need for a comprehensive taxonomic revision and a re-evaluation of the species boundaries among *F. celtidicola*, *F. juglandicola*, and *F. aconidiale*.

## 5 Conclusion

The results of this study provide new insights into the taxonomy of the FCCSC, demonstrating that the currently accepted separation among *F. aconidiale*, *F. celtidicola*, and *F. juglandicola* is not supported by molecular and distance-based evidence. Phylogenetic reconstruction, species delimitation analyses (ABGD, mPTP, and GMYC), and pairwise K2P distance comparisons consistently indicate that these taxa, together with the Italian and Polish isolates, form a single, genetically cohesive lineage characterized by shallow but structured intraspecific diversity. While ABGD and mPTP converged on a single species-level unit, GMYC detected limited substructure likely reflecting population-level differentiation or incomplete lineage sorting.

These findings highlight that, although the FCCSC is clearly distinct from the FTSC, internal diversification within the FCCSC remains below the interspecific thresholds typically recognized for *Fusarium*. The observed genetic cohesion suggests that several taxa currently regarded as separate species may instead represent variants of a single evolutionary lineage. More broadly, the study illustrates that while species boundaries in some *Fusarium* s.c. are robust and reproducible across methods, others remain ambiguous. This underscores the need to expand taxon sampling and adopt integrative approaches that combine molecular, morphological, and ecological data. In this respect, distance-based and model-based species delimitation algorithms provide a valuable framework for reassessing recently described taxa and for verifying the stability of species boundaries as the ongoing exploration of *Fusarium* diversity continues.

## Data Availability

The datasets presented in this study can be found in online repositories. The names of the repository/repositories and accession number(s) can be found at: https://www.ncbi.nlm.nih.gov/genbank/, several.

## Footnotes

1
http://tree.bio.ed.ac.uk/software/figtree/

2
https://inkscape.org
