# Supplementary figures and images for: Molecular investigation on hazelnut-associated Fusarium isolates belonging to the Fusarium citricola species complex
Source: Front Microbiol. 2026 Jan 9;16:1741069. doi: 10.3389/fmicb.2025.1741069 (PMC12827717; doi:10.3389/fmicb.2025.1741069)

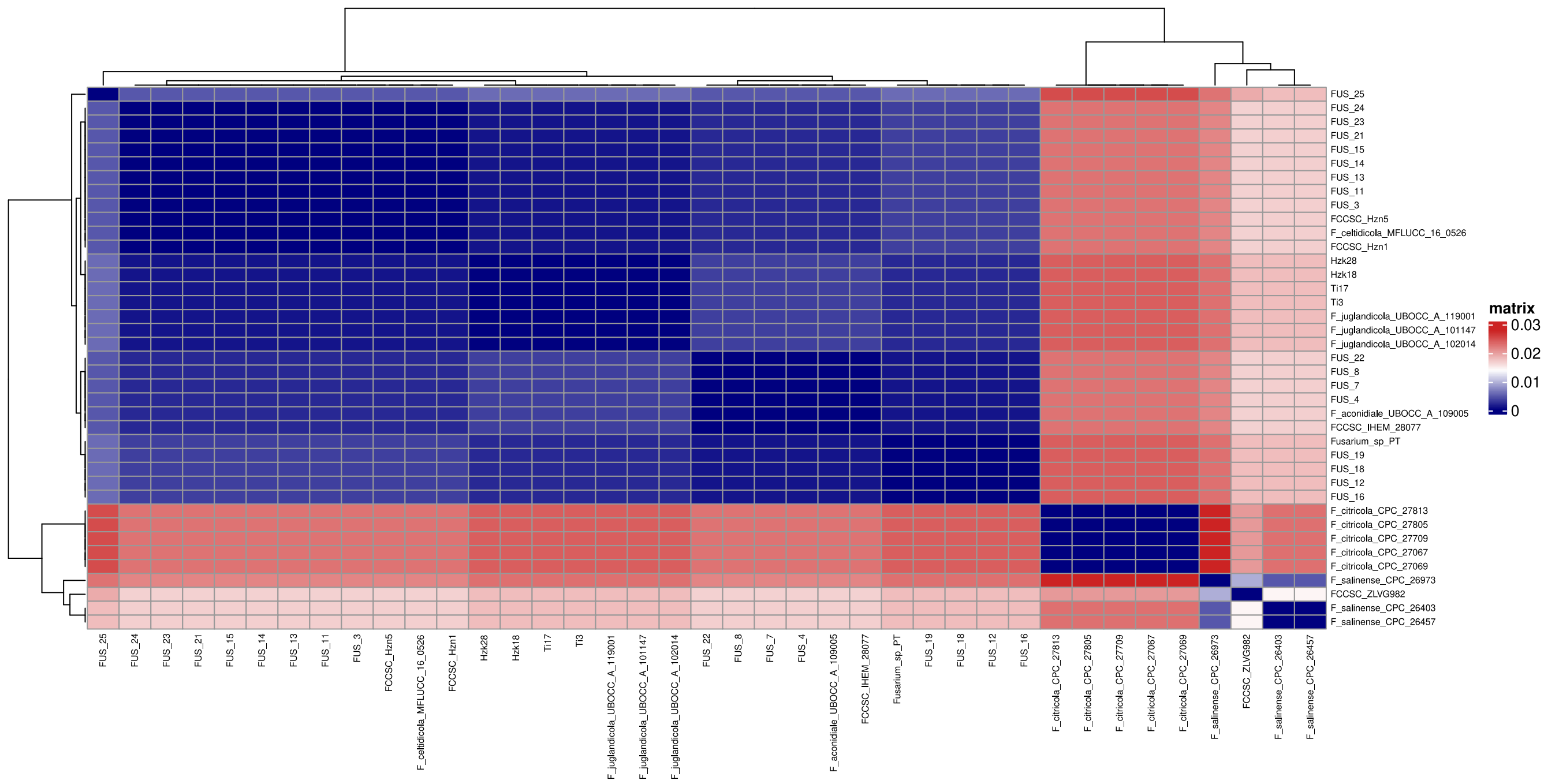

Supplement: Supplementary file 2 [file Image_2.pdf]
