# Supplementary material for: Molecular investigation on hazelnut-associated Fusarium isolates belonging to the Fusarium citricola species complex
Source: Front Microbiol. 2026 Jan 9;16:1741069. doi: 10.3389/fmicb.2025.1741069 (PMC12827717; doi:10.3389/fmicb.2025.1741069)
Supplement: Supplementary file 1 [file Image_1.pdf]

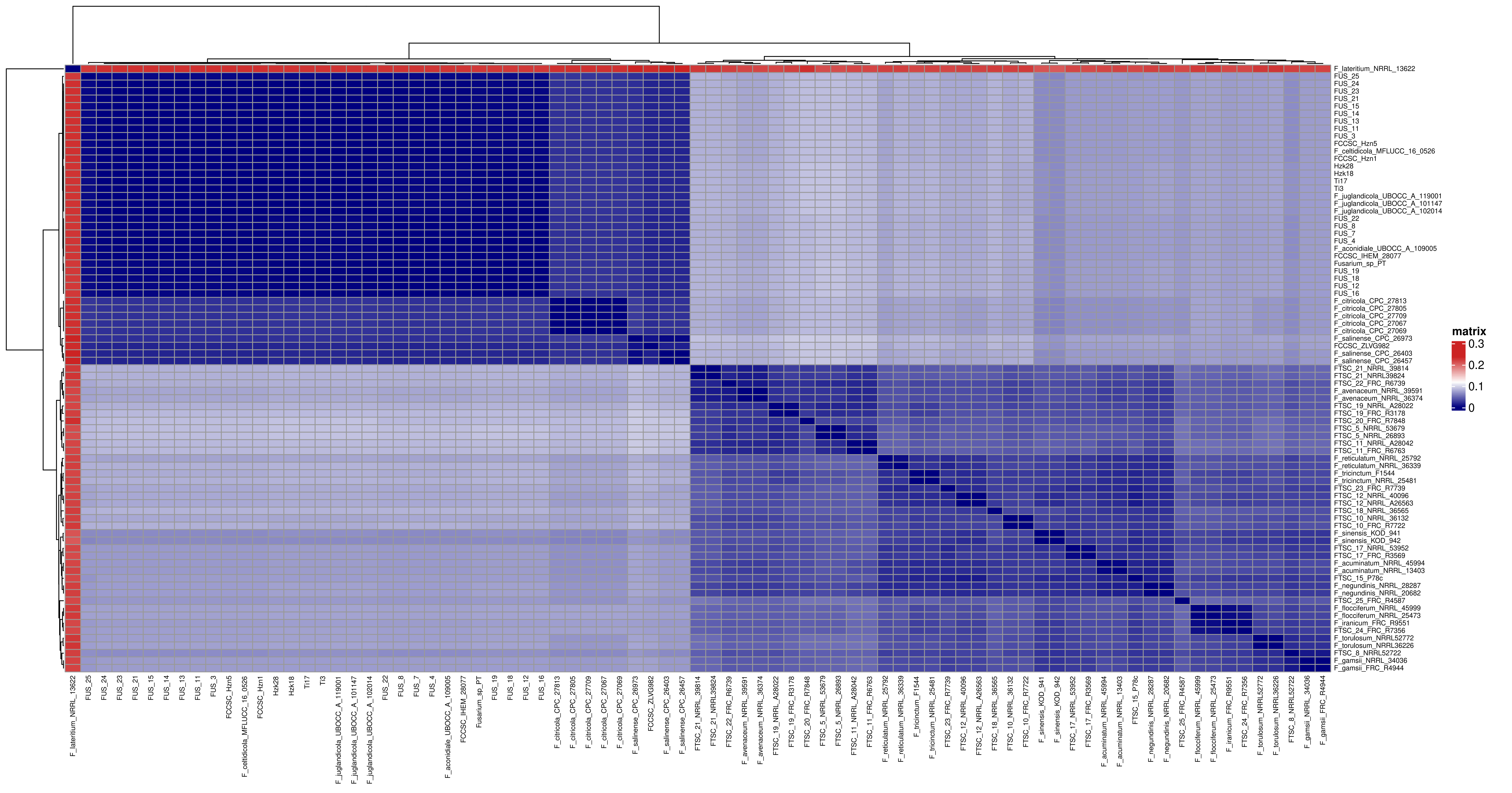
F_lateritium_NRRL_13622
FUS_25
FUS_24
FUS_23
FUS_21
FUS_15
FUS_14
FUS_13
FUS_11
FUS_3
FCCSC_Hzn5
F_celtidicola_MFLUCC_16_0526
FCCSC_Hzn1
Hzk28
Hzk18
Ti17
Ti3
F_juglandicola_UBOCC_A_119001
F_juglandicola_UBOCC_A_101147
F_juglandicola_UBOCC_A_102014
FUS_22
FUS_8
FUS_7
FUS_4
F_aconidiale_UBOCC_A_109005
FCCSC_IHEM_28077
Fusarium_sp_PT
FUS_19
FUS_18
FUS_12
FUS_16
F_citricola_CPC_27813
F_citricola_CPC_27805
F_citricola_CPC_27709
F_citricola_CPC_27067
F_citricola_CPC_27069
F_salinense_CPC_26973
FCCSC_ZLVG982
F_salinense_CPC_26403
F_salinense_CPC_26457
FTSC_21_NRRL_39814
FTSC_21_NRRL39824
FTSC_22_FRC_R6739
F_avenaceum_NRRL_39591
F_avenaceum_NRRL_36374
FTSC_19_NRRL_A28022
FTSC_19_FRC_R3178
FTSC_20_FRC_R7848
FTSC_5_NRRL_53679
FTSC_5_NRRL_26893
FTSC_11_NRRL_A28042
FTSC_11_FRC_R6763
F_reticulatum_NRRL_25792
F_reticulatum_NRRL_36339
F_tricinctum_F1544
F_tricinctum_NRRL_25481
FTSC_23_FRC_R7739
FTSC_12_NRRL_40096
FTSC_12_NRRL_A26563
FTSC_18_NRRL_36565
FTSC_10_NRRL_36132
FTSC_10_FRC_R7722
F_sinensis_KOD_941
F_sinensis_KOD_942
FTSC_17_NRRL_53952
FTSC_17_FRC_R3569
F_acuminatum_NRRL_45994
F_acuminatum_NRRL_13403
FTSC_15_P78c
F_negundinis_NRRL_28287
F_negundinis_NRRL_20682
FTSC_25_FRC_R4587
F_flocciferum_NRRL_45999
F_flocciferum_NRRL_25473
F_iranicum_FRC_R9551
FTSC_24_FRC_R7356
F_torulosum_NRRL52772
F_torulosum_NRRL36226
FTSC_8_NRRL52722
F_gamsii_NRRL_34036
F_gamsii_FRC_R4944
matrix
0.3
0.2
0.1
0
